# Change of Time of Issue

**Published:** 1889-01

**Authors:** 


### CHANGE OF TIME OF ISSUE.

It has been decided to change the date of issuing the journal to the fifteenth of the month. In the future the journal will be mailed regularly on the fourteenth day of the month, except when such date comes on the Sabbath, when it will be mailed on the preceding Saturday. We have been led to this change because of the fact that nearly all the dental journals published are issued upon the first of the month. We will be able to get news of interest which others that have gone to press earlier will not have; and, while on the other hand, they will have news that we will not get, yet such is our aim, and our desire is to publish news that cannot be found in other journals, and news also of such a character as will make the journal a necessity in the office of every progressive dentist. Send us in your subscription for 1889. We do not want to drop you from our list. The journal will come to you on the middle of the month, when you have gotten tired looking at the advertisements in the other journals, and will be a change that we know you will appreciate.

